# Posterior tibial slope has no impact on treatment outcome in anterior cruciate ligament revision patients

**DOI:** 10.1002/jeo2.70377

**Published:** 2025-08-29

**Authors:** Jacob Sorwad, Torsten Grønbech Nielsen, Ole Gade Sørensen, Lars Konradsen, Martin Lind

**Affiliations:** ^1^ Department of Sports Traumatology Aarhus University Hospital Aarhus N Denmark; ^2^ Section of Sports Traumatology, Bispebjerg and Frederiksberg University Hospital Copenhagen Denmark

**Keywords:** anterior cruciate ligament, Knee surgery, posterior tibial slope, Sports traumatology

## Abstract

**Purpose:**

To investigate the impact of posterior tibial slope (PTS) on postoperative outcome in an anterior cruciate ligament (ACL) revision cohort, based on sagittal knee stability and subjective, patient‐reported knee function.

**Methods:**

Lateral knee radiographs from 105 ACL revision patients (mean age 27.2 ± 6.5 years) were retrospectively reviewed and both medial and lateral posterior tibial slope was measured. Objective sagittal knee stability was based on Rolimeter measurements. The subjective knee function was obtained through the Knee Numeric‐Entity Evaluation Score (KNEES‐ACL), the Knee Injury and Osteoarthritis Outcome Score (KOOS) and Tegner Activity Scale (TAS) questionnaires. Objective anterior–posterior (AP) knee laxity was examined prior to ACL revision surgery and at a one‐year follow‐up, and the patient reported outcome measures (PROMs) were obtained prior to ACL revision surgery and after a two‐year follow‐up period.

**Results:**

No correlation was found between medial PTS and knee stability before (0.16; 95% confidence interval [CI], −0.06 to 0.36, *p* = 0.15) or one year after ACL revision surgery (0.07; 95% CI, −0.14 to 0.27, *p* = 0.54). Likewise, no correlation was found between lateral PTS and knee stability before (0.30; 95% CI, 0.09–0.48, *p* = 0.01) and one year after ACL revision surgery (0.15; 95% CI, −0.06 to 0.35, *p* = 0.16). Likewise, there was no correlation between medial and lateral PTS and KOOS, KNEES‐ACL and TAS. The mean lateral PTS was 2.6° steeper than the medial PTS (*p* < 0.05).

**Conclusion:**

In the present study, PTS was not found to be associated with either sagittal knee stability or subjective knee function in ACL revision patients. Patients undergoing ACL revision surgery have a large mean difference between the medial and the lateral PTS.

**Level of Evidence:**

Level IV.

**Abbreviations**
ACL: anterior cruciate ligament
ACLR: anterior cruciate ligament reconstruction
ALL: anterolateral ligament
AP: anterior‐posterior
ATT: anterior tibial translation
ICC: inter‐/intra‐observer correlation coefficient
IKDC: international knee documentation committee
KNEES‐ACL: knee numeric‐entity evaluation score
KOOS: knee injury and osteoarthritis outcome score
MRI: magnetic resonance imaging
PROM: patient reported outcome measure
PTS: posterior tibial slope
SD: standard deviation
TAS: tegner activity scale questionnaires
TPAA: tibial proximal anatomical axis

## INTRODUCTION

Several studies have tried to identify essential risk factors related to anterior cruciate ligament (ACL) injuries [1, 9, 31]. The inclination in the sagittal plane of the tibial plateau, known as the posterior tibial slope (PTS), has been associated with the occurrence of ACL injuries [3, 5, 12, 15, 17, 26]. Therefore, PTS has increasingly gained attention from knee surgeons and sports medicine researchers. Studies have found a significant association between increased PTS and anterior tibial translation (ATT), resulting in increased ACL‐tissue load [12, 15]. Christensen et al. reported significantly greater strain on an ACL graft as a result of an increase of two degrees in the PTS [3]. Todd et al. compared PTS in a group with a non‐contact ACL injury and a control group with no history of ACL injury, finding an increased PTS in the ACL injury group compared to the control group [26]. Todd et al. found increased lateral PTS to be a significant risk factor for early graft failure after ACL reconstruction [26]. Also, Imhoff et al. found that a decreased PTS could have a protective effect on the ACL graft in ACL revision patients [12]. Both Napier et al. and Engler et al. found a significantly increased PTS in patients with ACL graft failure after primary anterior cruciate ligament reconstruction (ACLR) compared to non‐failure patients who had ACLR [6, 17].

Finally, a systematic review and meta‐analysis including 82 studies and 12,971 patients concluded that patients with ACLR graft failure have an increased lateral and medial PTS compared to patients with intact ACLR or native ACL [5].

However, the literature differs when it comes to the association between PTS and the clinical outcome (sagittal stability and subjective knee function) after primary ACLR. Batty et al. suggested that an increased PTS was associated with high‐grade pivot shift and instability but that there was no association with patient‐reported outcome measurements (PROMs) [2]. Similarly, Yoon et al. found no association between PTS and subjective outcome scores (International Knee Documentation Committee [IKDC] [11], Lysholm [24] and Tegner Activity Scale [TAS] [24]) and between PTS and knee stability (Rolimeter and side‐to‐side difference in Telos stress radiographs) in primary ACLR patients [33]. Winkler et al. found that patients with multiple ACL graft failures had significantly worse PROM‐scores, higher PTS or higher re‐surgery rates and complication compared to patients with primary ACL graft failure [31].

The literature regarding the impact of PTS on sagittal knee stability and subjective knee function in ACL revision patients is very limited. Therefore, the purpose of this study was to investigate the impact of PTS regarding sagittal knee stability and PROMs on postoperative outcomes in an ACL revision cohort. However, it has been described, that patients with a PTS of >12° has a higher risk of ACL‐injuries, hence a higher revision rate—known as the ‘12 degrees rule’ [19, 29].

Therefore, it was hypothesized that a PTS > 12° would result in increased knee laxity and affect subjective knee function resulting in lower PROM‐scores in ACL revision patients.

## METHODS

### Study design and participants

The study design was a retrospective cohort study. The patient data originated from a randomized study with 110 patients, in which the impact of supplemental reconstruction of the anterolateral ligament (ALL) combined with ACL revision was investigated [22]. Inclusion criteria consisted of patients aged 18–50 years with an ACL‐deficient knee scheduled for their first ACL revision. Both Stage 1 and Stage 2 ACL revisions were included. Exclusion criteria included language barriers, concurrent instability of the collateral or posterior cruciate ligaments, concomitant procedures such as meniscus/cartilage transplant and concomitant osteotomy. No association between reconstruction of the ALL, postoperative stability and subjective, patient‐reported knee function was found [22]. Therefore, the study cohort outcome could be used to investigate other factors that could affect outcome. PTS measurements required tibial radiographs with a minimum of 10 centimeters of tibia visualized. An exclusion criterion was a missing lateral knee radiograph or a lateral knee radiograph with a tibial cut‐off less than 10 centimeters from the joint line. All radiographs were obtained prior to revision ACLR.

The objective anterior–posterior (AP) knee laxity was examined at a 1‐year follow‐up, and the PROMs were obtained after a 2‐year follow‐up period.

To investigate, whether a PTS > 12° would result in increased knee laxity and affect subjective knee function, two cohorts were created. Patients were divided into groups making it possible to differentiate between patients with PTS > and < than 12° on both medial and lateral side.

### Measurement of knee stability

The objective AP knee laxity was measured with the knee being in 20° of flexion by an independent physiotherapist at maximal knee translation in the sagittal plane, using a Rolimeter [8] developed by Aircast Europe (Neubeuern, Germany). The laxity measurements were performed prior to and one year after ACL‐revision surgery.

### Patient‐reported outcome measures (PROMs)

The postoperative subjective knee function was based on two PROMs: the Knee Numeric‐Entity Evaluation Score (KNEES–ACL) [4] (including activities of daily living, psychosocial aspects, looseness, slackness and sport and recreation) and the Knee Injury and Osteoarthritis Outcome Score (KOOS) [18] (including symptoms, pain, daily living, sports, recreational living and quality of life). Furthermore, the postoperative knee function was based on the function scale, the Tegner activity scale [24]. All PROMs were obtained prior to and two years after ACL‐revision surgery.

### Measurement of PTS

**Figure 1:**
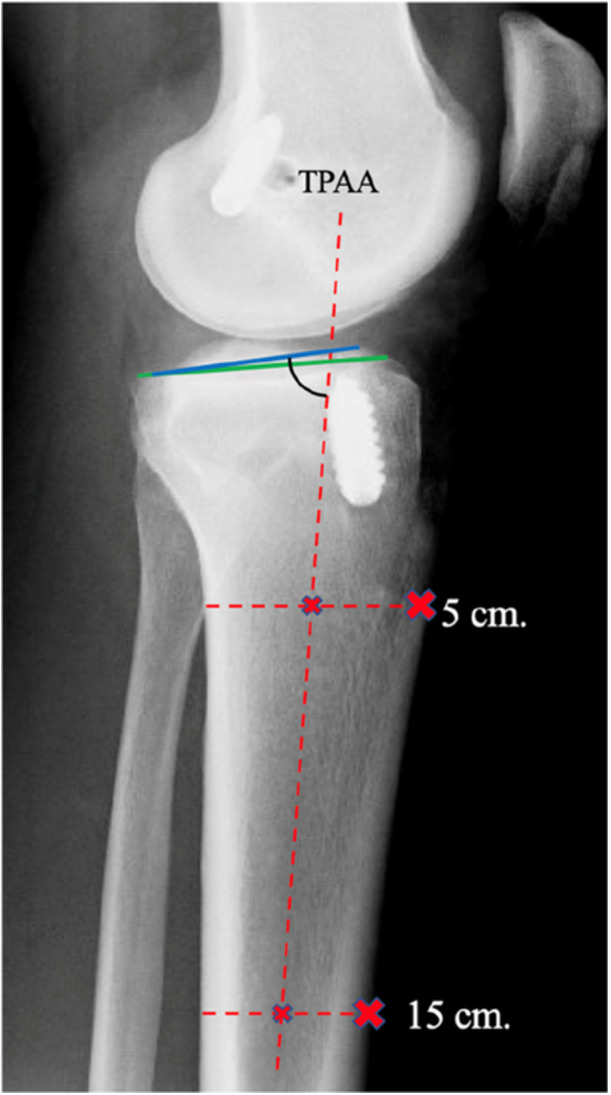
Method of measuring medial and lateral posterior tibial slope. TPAA, tibial proximal anatomical axis.

The measurements of the PTS were made using the computer software program IMPAX Client (version 6.5) developed by Agfa HealthCare (Mortsel, Belgium). The measurements were carried out according to an earlier described method for this purpose [27]. The reference axis in this present study was chosen to be the tibial proximal anatomical axis (TPAA) (Figure 1). This reference axis has been shown to precisely represent the mechanical tibial axis when using short lateral knee radiographs for measurement [32].

Lines were drawn 5 cm and 15 cm distal from the anteromedial surface of the tibial plateau to the anterior cortex. Transverse lines were drawn to connect the anterior and posterior tibial cortices at the shortest distance. The midpoint of the transverse lines was marked, and an intersecting line, the TPAA, was drawn (Figure 1).

The degree of the tibial slope was measured between the TPAA and both the medial and the lateral tibial plateau inclination angles. The final tibial slope was retrieved by subtracting the measured grade from 90. In some of the radiographs, the distal distance from the anteromedial joint surface of the tibial plateau was shorter than 15 cm. Therefore, the distal mark on the anterior cortex was made 10 cm distally from the anteromedial joint surface of the tibial plateau instead.

All measurements were performed twice, the second measurement being blinded from the first measurement to enable intraobserver precision calculations. Furthermore, a random selected group of radiographs was measured by two different physicians to ensure that interobserver precision calculations could be done.

### Statistical analysis

All statistical calculations were done using the software program Stata version 17 developed by StataCorp (College Station, TX). The clinically relevant difference for sagittal knee laxity was set to 1.0 mm with a standard deviation (SD) of 1.5 mm based on clinical experience of the authors. The alpha‐value was set to 0.05, and the beta‐value was set to 0.20. To achieve this power, the study would require at least 70 patients. Correlation tests were made between PROMs/TAS/knee stability and medial/lateral PTS. In addition, a dichotomization of the slope measurements (above or equal to/below 12°) for medial and lateral PTS was made as an increased PTS results in a biomechanical greater strain on the ACL graft [12, 15]. Furthermore, a PTS > 12° has shown to be a significant risk factor of ACL‐injuries [19, 29].

A student's *t*‐test was conducted to compare the groups based on KNEES sub‐scores (slackness and looseness) and sagittal knee stability.

All measurements were analysed to calculate the intraobserver correlation coefficient (ICC) mean difference ± two standard deviations. A study population sample (*n* = 20) went through independent analysis to calculate the interobserver correlation coefficient (ICC) between the observers.

## RESULTS

Of the 110 patients, lateral knee radiographs were unavailable for five individuals. Lateral knee radiographs from the remaining 105 patients who underwent ACL revision surgery from 2016 to 2019 were retrospectively reviewed and measured. Nine patients were excluded owing to insufficient length of the lateral knee radiograph. The final cohort included a total of 96 patients, 46 males and 50 females (mean age 27.2 ± 6.5 years). The baseline demographics of included patients appears in Table 1 (after dichotomization) and Table 2. The mean time between primary ACLR and revision ACLR was 55.8 months ± 41.2 months (mean ± SD). The mean medial and lateral PTSs were 9.2° ± 2.6 (mean ± SD) and 11.8° ± 2.6 (mean ± SD), respectively. This difference was found to be statistically significant (*p* < 0.001).

**Table 1:** Baseline demographics of included patients after dichotomization.

**Medial posterior tibial slope**			
Dichotomous division	<12°	>12°	
No. of patients	84	12	
			*p* value^a^
Age (years), mean ± SD	26.8 ± 6.4	29.5 ± 6.8	0.18
Sex (male), *n* (%)	44 (52.4)	6 (50.0)	0.88
Body mass index (kg/m^2^), mean ± SD	24.4 ± 3.2	24.7 ± 3.3	0.76
Injury mechanism			
Sport, *n* (%)	41 (48.8)	4 (33.3)	0.46
Other, *n* (%)	28 (33.3)	6 (50.0)	
Unknown, *n* (%)	15 (17.9)	2 (16.7)	
Additional anterolateral ligament, *n* (%)	42 (52.6)	5 (41.7)	0.56
Meniscus injury‐rev., *n* (%)	27 (32.5)	6 (50)	0.24
Medial, *n* (%)	15 (18.1)	2 (16.7)	
Resection, *n* (%)	9 (10.8)	2 (16.7)	
Repair, *n* (%)	6 (7.2)	0 (0)	
Lateral, *n* (%)	17 (20.5)	4 (33.3)	
Resection, *n* (%)	13 (15.7)	2 (16.7)	
Repair, *n* (%)	4 (4.8)	2 (16.7)	
Cartilage injury‐rev., *n* (%)	39 (47.6)	5 (41.7)	0.70
Debridement, *n* (%)	2 (2.4)	0 (0)	
Untouched, *n* (%)	37 (45.2)	5 (41.7)	
**Lateral posterior tibial slope**			
Dichotomous division	<12°	>12°	
No. of patients	51	45	
			*p* value^a^
Age (years), mean ± SD	27.6 ± 6.3	26.6 ± 6.6	0.43
Sex (male), *n* (%)	33 (64.7)	17 (37.8)	0.01
Body mass index (kg/m^2^), mean ± SD	24.4 ± 3.1	24.4 ± 3.2	1.00
Injury mechanism			
Sport, *n* (%)	25 (49.0)	20 (45.5)	0.85
Other, *n* (%)	17 (34.3)	17 (38.6)	
Unknown, *n* (%)	9 (17.7)	7 (15.9)	
Additional anterolateral ligament, *n* (%)	23 (45.1)	24 (54.6)	0.84
Meniscus injury‐rev., *n* (%)	17 (33.3)	16 (36.4)	0.76
Medial, *n* (%)	8 (15.7)	9 (20.5)	
Resection, *n* (%)	5 (9.8)	6 (13.6)	
Repair, *n* (%)	3 (5.9)	3 (6.8)	
Lateral, *n* (%)	12 (23.5)	9 (20.5)	
Resection, *n* (%)	7 (13.7)	6 (13.6)	
Repair, *n* (%)	5 (9.8)	3 (6.8)	
Cartilage injury‐rev., *n* (%)	21 (42.0)	23 (52.3)	0.32
Debridement, *n* (%)	2 (4.0)	0 (0.0)	
Untouched, *n* (%)	19 (38.0)	23 (52.3)	

Abbreviations: rev., at revision surgery; SD, standard deviation.

^a^ Testing value between the groups.

**Table 2:** Baseline demographics of included patients.

Patient demographics	
No. of patients	96
Age (years), mean ± SD	27.2 ± 6.5
Sex (male), *n* (%)	50 (52)
Body mass index (kg/m^2^), mean ± SD	24.4 ± 3.2
ACL revision graft type	
BPTB, *n* (%)	46 (48)
QTB, *n* (%)	26 (27)
STG, *n* (%)	7 (7)
Allograft, *n* (%)	16 (17)
Injury mechanism	
Sport, *n* (%)	45 (47)
Other, *n* (%)	34 (46)
Unknown, *n* (%)	16 (17)
Additional anterolateral ligament, *n* (%)	47 (49)
Meniscus injury‐rev., *n* (%)	33 (35)
Medial, *n* (%)	17 (18)
Resection, *n* (%)	11 (11)
Repair, *n* (%)	6 (6)
Lateral, *n* (%)	21 (22)
Resection, *n* (%)	15 (16)
Repair, *n* (%)	6 (6)
Cartilage injury‐rev., *n* (%)	44 (46)
Debridement, *n* (%)	2 (2)
Untouched, *n* (%)	42 (44)

Abbreviations: ACL, anterior cruciate ligament; BPTB, bone–patellar tendon–bone; QTB, quadriceps tendon‐bone; rev., at revision surgery; SD, standard deviation; STG, semitendinosus + gracilis.

Intraobserver reliability for PTS measurements demonstrated excellent agreement (medial slope, ICC = 0.96 [95% CI, 0.93–0.97]; lateral slope, ICC = 0.95 [95% CI, 0.93–0.97]). Likewise, the ICC interobserver analysis demonstrated good correlation (medial slope, ICC = 0.76 [95% CI, 0.48–0.90]; lateral slope, ICC = 0.78 [95% CI, 0.52–0.91]).

The mean differences ± SDs between the first and second measurements were 0.10° ± 0.90° (mean ± SD) and −0.01° ± 0.90° (mean ± SD) for the medial and lateral PTS, respectively. This indicates high intraobserver repeatability between the first and second measurements.

No correlation between medial PTS and sagittal knee stability before revision surgery (0.16; 95% CI, −0.06 to 0.36, *p* = 0.15) and one year after revision surgery (0.07; 95% CI, −0.14 to 0.27, *p* = 0.54) was found. The same applies to the correlation between lateral PTS and sagittal knee stability before revision surgery (0.30; 95% CI, 0.09–0.48, *p* = 0.01) and 1 year after revision surgery (0.15; 95% CI, −0.06 to 0.35, *p* = 0.16).

There was no correlation between KOOS and medial and lateral PTS before revision surgery and two years after revision surgery (Table 3).

**Table 3:** Correlation analysis between Knee injury and Osteoarthritis Outcome Score and posterior tibial slope.

	Correlation	95% CI (Lower)	95% CI (Upper)	*p* value
**Medial posterior tibial slope**				
Knee injury and Osteoarthritis Outcome Score (before ACL revision)				
Symptoms	0.08	−0.13	0.28	0.46
Pain	−0.10	−0.30	0.11	0.35
Function, daily living	−0.10	−0.30	0.11	0.34
Function, sports and recreation	−0.01	−0.22	0.20	0.91
Quality of life	0.03	−0.18	0.24	0.76
Knee injury and Osteoarthritis Outcome Score (two years after ACL revision)				
Symptoms	0.19	−0.04	0.40	0.11
Pain	0.11	−0.12	0.33	0.36
Function, daily living	0.09	−0.14	0.31	0.45
Function, sports and recreation	−0.02	−0.25	0.30	0.87
Quality of life	0.08	−0.15	0.30	0.50
**Lateral posterior tibial slope**				
Knee injury and Osteoarthritis Outcome Score (before ACL revision)				
Symptoms	0.003	−0.20	0.21	0.98
Pain	−0.19	−0.38	0.02	0.08
Function, daily living	−0.18	−0.37	0.03	0.10
Function, sports and recreation	−0.15	−0.35	0.06	0.16
Quality of life	−0.11	−0.31	0.10	0.30
Knee injury and Osteoarthritis Outcome Score (two years after ACL revision)				
Symptoms	0.04	−0.19	0.27	0.73
Pain	−0.07	−0.29	0.16	0.56
Function, daily living	−0.06	−0.28	0.17	0.63
Function, sports and recreation	−0.13	−0.34	0.10	0.29
Quality of life	−0.10	−0.32	0.14	0.42

Abbreviations: ACL, anterior cruciate ligament; CI, confidence interval.

KNEES‐ACL had no significant correlation with medial or lateral PTS before revision surgery or two years after revision surgery (Table 4).

**Table 4:** Correlation analysis between Knee Numeric‐Entity Evaluation Score and posterior tibial slope.

	Correlation	95% CI (Lower)	95% CI (Upper)	*p* value
**Medial posterior tibial slope**				
Knee Numeric‐Entity Evaluation Score (before ACL revision)				
Activity of daily living	0.07	−0.14	0.27	0.51
Psychosocial	−0.03	−0.14	0.17	0.76
Looseness	−0.04	−0.25	0.17	0.70
Slackness	0.03	−0.18	0.23	0.80
Symptoms	−0.07	−0.27	0.14	0.54
Sport and recreation, behaviour	−0.10	−0.30	0.17	0.38
Sport and recreation, physical	−0.08	−0.31	0.17	0.54
Knee Numeric‐Entity Evaluation Score (two years after ACL revision)				
Activity of daily living	−0.04	−0.27	0.18	0.71
Psychosocial	−0.15	−0.36	0.08	0.21
Looseness	−0.07	−0.30	0.16	0.54
Slackness	−0.08	−0.30	0.15	0.51
Symptoms	−0.08	−0.30	0.15	0.51
Sport and recreation, behaviour	−0.10	−0.33	0.13	0.39
Sport and recreation, physical	−0.01	−0.25	0.23	0.93
**Lateral posterior tibial slope**				
Knee Numeric‐Entity Evaluation Score (before ACL revision)				
Activity of daily living	0.16	−0.05	0.35	0.14
Psychosocial	0.07	−0.14	0.28	0.49
Looseness	0.15	−0.06	0.35	0.16
Slackness	0.14	−0.07	0.34	0.19
Symptoms	0.07	−0.14	0.27	0.50
Sport and recreation, behaviour	0.07	−0.15	0.35	0.54.
Sport and recreation, physical	0.03	−0.21	0.26	0.93
Knee Numeric‐Entity Evaluation Score (two years after ACL revision)				
Activity of daily living	0.16	−0.07	0.37	0.17
Psychosocial	−0.09	−0.31	0.14	0.42
Looseness	0.14	−0.09	0.36	0.22
Slackness	0.07	−0.16	0.29	0.55
Symptoms	0.07	−0.15	0.30	0.53
Sport and recreation, behaviour	0.07	−0.17	0.29	0.57
Sport and recreation, physical	0.19	−0.05	0.41	0.13

Abbreviations: ACL, anterior cruciate ligament; CI, confidence interval.

In addition, no correlation between medial PTS and TAS before ACL revision surgery (0.00; 95% CI, −0.21 to 0.21, *p* = 0.99) and 2 years after revision surgery (0.07; 95% CI, −0.16 to 0.29, *p* = 0.56) was found. Likewise, there was no correlation between lateral PTS and TAS before ACL revision surgery (−0.01; 95% CI, −0.21 to 0.20, *p* = 0.98) and 2 years after revision surgery (−0.11; 95% CI, −0.33 to 0.12, *p* = 0.37).

The dichotomisation of the data, dividing the medial and lateral PTS measurements into two groups, each based on a cut‐off point at 12°, did not yield any correlation between PTS and sagittal knee stability either before revision surgery or one year after surgery (Table 5).

**Table 5:** *T*‐test after dichotomous division (>/≤ 12°) of the medial and lateral posterior tibial slope measurements.

**Medial posterior tibial slope**			
Dichotomous division	<12°	>12°	
No. of patients	84	12	
	Mean (95% CI)	Mean (95% CI)	*p* value^a^
Posterior tibial slope, (grades)	**8.5 (8.1–9.0)**	**13.8 (13.3–14.4)**	**<0.01**
Knee stability, (mm)^b^ (before ACL revision surgery)	5.4 (4.8–5.9)	5.6 (3.6–7.6)	0.75
Knee stability, (mm)^b^ (1 year after ACL revision surgery)	2.1 (1.7–2.5)	2.3 (1.1–2.9)	0.41
KNEES‐ACL subscore (before ACL revision surgery)			
Slackness (points)	9.5 (8.4–10.6)	10.6 (6.6–14.7)	0.49
Looseness (points)	5.8 (5.0–6.6)	5.6 (2.8–8.5)	0.85
KNEES‐ACL subscore (2 years after ACL revision surgery)			
Slackness (points)	7.9 (6.7–9.0)	7.4 (2.5–12.3)	0.80
Looseness (points)	3.8 (3.0–4.5)	3.6 (0.2–6.9)	0.85
**Lateral posterior tibial slope**			
Dichotomous division	<12°	>12°	
No. of patients	51	45	
	Mean (95% CI)	Mean (95% CI)	*p* value^a^
Posterior tibial slope, (grades)	**9.9 (9.5–10.3)**	**14.0 (13.5–14.5)**	**<0.01**
Knee stability, (mm)^b^ (before ACL revision surgery)	4.9 (4.4–5.4)	6.0 (5.0–6.9)	0.03
Knee stability, (mm)^b^ (1 year after ACL revision surgery)	1.9 (1.4–2.4)	2.2 (1.6–2.8)	0.42
KNEES‐ACL subscore (before ACL revision surgery)			
Slackness (points)	8.7 (7.2–10.2)	10.7 (9.1–12.2)	0.07
Looseness (points)	5.2 (4.1–6.3)	6.3 (5.2–7.5)	0.14
KNEES‐ACL subscore (2 years after ACL revision)			
Slackness (points)	7.5 (5.9–9.1)	8.2 (6.5–9.1)	0.57
Looseness (points)	3.5 (2.6–4.4)	4.0 (2.8–5.2)	0.51

Abbreviations: ACL, anterior cruciate ligament; CI, confidence interval; KNEES‐ACL, Knee Numeric‐Entity Evaluation Scores.

^a^ Testing value between the groups.

^b^ Anterior tibial translation measured on Rolimeter.

In addition, no significant association between PTS and KNEES sub‐scores was found, either before revision surgery or two years after surgery.

During the 2‐year follow period, 3 of the included patients sustained a third ACL‐injury.

The PTS‐measurements on these patients were as follows: 10°/12°, 5.7°/8.5°, and 9.95°/12.85° (medial PTS/lateral PTS) respectively.

## DISCUSSION

The primary finding of the present study was that there was no significant association between PTS and sagittal knee stability and PROMs in first time ACL revision patients.

Existing literature dealing with PTS and PROMs in relation to ACL revision surgery is limited. Yoon et al. [34] found no intergroup difference in PROMs between ACL revision and ACL re‐revision patients, based on IKDC [11], Lysholm [24] and TAS [24]. The mean PTSs for ACL revision and ACL re‐revision patients were 11.5° ± 3.6° and 13.1° ± 2.4°, respectively. On the other hand, the study found a greater knee laxity in the ACL re‐revision group at two‐year follow‐up. Batty et al. [2] suggested that high‐grade pivot shift and knee instability were associated with PTS > 9°. Nevertheless, PTS had no association with any of the PROMs included in the study (four‐item Pain Intensity Measure, ACL Quality of Life questionnaire, IKDC and KOOS). Likewise, Yoon et al. [33] found no association between PTS and the PROMs included in their study (IKDC, Lysholm and TAS). Also, no association between PTS and knee stability was found.

The present study demonstrated as a secondary finding that the mean lateral PTS (11.8° ± 2.6°) was 2.6° increased compared to the mean medial PTS (9.2° ± 2.6°). Only a few studies on PTS values in ACLR revision patients exist. Napier et al. [17] found a similar result in revision ACLR patients with a mean PTS difference of 5.6° (medial PTS: 6.3° ± 2.7° and lateral PTS: 11.9° ± 3.0°) and an even higher mean PTS difference in third‐time ACL‐injury patients, at 6.1° (medial PTS: 7.5° ± 3.0° and lateral PTS: 13.6° ± 3.1°). The present study supports that finding. Dean et al. [5] found a significantly larger lateral and medial PTS in a group with revision ACLR graft failure compared to both a group with a primary ACL graft failure and a group with an intact ACL. The mean differences between medial and lateral PTS were only 0.5°, 0.89° and 0.71°, respectively. However, Christensen et al. [3] compared a group with ACLR graft failure and a group with ACLR without graft failure and found lateral PTS to be a significant risk factor for early graft failure.

Furthermore, Stijak et al. [23] investigated medial and lateral PTS in ACL‐deficient knees, the measurements of PTS being carried out using magnetic resonance imaging (MRI). The study suggested lateral PTS to be a more significant risk factor for ACL injuries compared with medial PTS. In addition, McLean et al. [16] indicated that an increased lateral PTS could potentially result in a greater anterior translation of the lateral compartment, creating an internal rotation of the tibia, hence a potentially higher ACL graft‐load force. Furthermore, the lateral PTS was significantly correlated to peak anterior knee joint reaction force [16]. This finding was supported by other studies dealing with knee biomechanics in relation to ACL injuries and ACL graft loads [7, 14, 20]. However, although these previous findings suggest that increased PTS could result in increased knee laxity, the present study found no association between PTS (both medial and lateral) and sagittal knee stability.

According to Utzschneider et al. [27], a true lateral view is needed to examine and measure the PTS correctly using conventional radiographs. The study suggests that, if true lateral knee imaging with a rotation of 0° is obtained, the method does not differ from measurements carried out using computed tomography, MRI or manual measurement. Rotation of the tibia bone, both external and internal, results in both increased medial and lateral PTS. However, the mean difference between medial and lateral PTS seems to decrease as the rotation increases in both directions [27, 28].

In contrast, Lee et al. [13] found an increased PTS in lateral radiographs compared to MRI, but even though sagittal‐plane MRI was previously considered superior to conventional radiography, the literature presents divergent results regarding measurements of PTS using MRI [21, 25].

True lateral radiographs were not guaranteed in our report. This could have influenced the steepness of the measurements, resulting in an overestimation of both medial and lateral PTS.

A limitation of the study was its sample size and the study‐type itself (retrospective), which means that the findings cannot be used to draw conclusions about the impact of PTS on revisions or graft failure risk. The data originates from a prior study evaluating a different primary outcome.

The follow‐up time was 1 and 2 years, and a longer period of follow‐up might result in other conclusions. Therefore, this is a limitation of the present study.

It was difficult to distinguish the medial and lateral parts of the tibial plateau on lateral radiographs, this being a two‐dimensional measurement because the radiographs are superimposed [10]. This combined with differential rotation of the knee radiographs was one of the present study's limitations.

## CONCLUSION

In the present study, PTS was not found to be associated with either sagittal knee stability or PROMs outcomes in patients undergoing ACL revision surgery. A secondary anatomical finding was that patients undergoing ACL revision surgery exhibited a large difference between medial and lateral PTS.

## AUTHOR CONTRIBUTIONS

Jacob Sorwad: Data extraction, data analyses, manuscript preparation. Martin Lind: Study design, patient surgery and follow‐up, data analyses, manuscript preparation. Ole Gade Sørensen: Study design, patient surgery and follow‐up, data analyses, manuscript editing. Torsten Grønbech Nielsen: Patient follow‐up, data analyses, manuscript editing. Lars Konradsen: Patient surgery and follow‐up, manuscript editing.

## CONFLICT OF INTEREST STATEMENT

The authors declare no conflicts of interest.

## ETHICS STATEMENT

Informed consent was obtained from all individual participants included in the study.This retrospective cohort study involving human participants was in accordance with the ethical standards of the institutional and national research committee. The study received approval from the Danish Ethical Committee (1‐10‐72‐324‐15) and was registered on ClinicalTrials.gov (NCT02680821).

## Data Availability

Data are not available due to GDPR restrictions.
